# Anemia detection through non-invasive analysis of lip mucosa images

**DOI:** 10.3389/fdata.2023.1241899

**Published:** 2023-10-19

**Authors:** Shekhar Mahmud, Turker Berk Donmez, Mohammed Mansour, Mustafa Kutlu, Chris Freeman

**Affiliations:** ^1^Department of Systems Engineering, Military Technological College, Muscat, Oman; ^2^Department of Biomedical Engineering, Sakarya University of Applied Sciences, Serdivan, Sakarya, Türkiye; ^3^Department of Mechatronics Engineering, Sakarya University of Applied Sciences, Serdivan, Sakarya, Türkiye; ^4^Electronics and Computer Sciences, University of Southampton, Southampton, United Kingdom

**Keywords:** anemia, machine learning, classification, support vector machine (SVM), decision tree

## Abstract

This paper aims to detect anemia using images of the lip mucosa, where the skin tissue is thin, and to confirm the feasibility of detecting anemia noninvasively and in the home environment using machine learning (ML). Data were collected from 138 patients, including 100 women and 38 men. Six ML algorithms: artificial neural network (ANN), decision tree (DT), k-nearest neighbors (KNN), logistic regression (LR), naive bayes (NB), and support vector machine (SVM) which are widely used in medical applications, were used to classify the collected data. Two different data types were obtained from participants' images (RGB red color values and HSV saturation values) as features, with age, sex, and hemoglobin levels utilized to perform classification. The ML algorithm was used to analyze and classify images of the lip mucosa quickly and accurately, potentially increasing the efficiency of anemia screening programs. The accuracy, precision, recall, and F-measure were evaluated to assess how well ML models performed in predicting anemia. The results showed that NB reported the highest accuracy (96%) among the other ML models used. DT, KNN and ANN reported an accuracies of (93%), while LR and SVM had an accuracy of (79%) and (75%) receptively. This research suggests that employing ML approaches to identify anemia will help classify the diagnosis, which will then help to create efficient preventive measures. Compared to blood tests, this noninvasive procedure is more practical and accessible to patients. Furthermore, ML algorithms may be created and trained to assess lip mucosa photos at a minimal cost, making it an affordable screening method in regions with a shortage of healthcare resources.

## Introduction

Anemia is characterized by a reduction of hemoglobin-containing red blood cells in the blood. The criteria for anemia, as determined by the World Health Organization (WHO), are defined as a hemoglobin level in the blood below 13 g/dL in men, 12 g/dL in women, and below 11 g/dL in pregnant women (Conrad, 2011). The most common causes of anemia are a decrease in red blood cell production or an increase in red blood cell destruction and loss, which is higher than normal (Brown, 1991; Aapro et al., 2002; Martinsson et al., 2014). Additionally, the production of malformed red blood cells in some hereditary blood diseases can also cause anemia. This results in a decrease in the average red blood cell count in the blood. The gold standard for detecting anemia is by taking intravenous blood from a venous vein and analyzing this blood by hemogram (Prefumo et al., 2019; An et al., 2021; Milovanovic et al., 2022). However, invasive procedures, particularly in pregnant and pediatric groups, are painful and difficult to coordinate (Bashiri et al., 2003). The subject must also go to a clinic to receive the relevant procedure. In light of the coronavirus pandemic that began in 2019, performing these procedures in medicine and conventional follow-up mechanisms are no longer feasible. Non-invasive anemia follow-up may provide benefits in terms of patient comfort. Although anemia has many clinical side effects, it typically progresses with pallor of the skin. Therefore, it is more easily diagnosed in areas where the skin is thin, such as the conjunctiva, lips, tongue, and oral mucosa were Wang et al. employed a smartphone application that uses the camera and various illumination sources to noninvasive check blood hemoglobin content, Tamir et al. identified anemia by examining the pallor of the eye's front conjunctiva, by examining the color and metadata of smartphone photographs taken on the fingernail bed, Mannio et al. estimate hemoglobin levels and Selfie Anemia, a non-invasive hemoglobin estimate smartphone app that operates under regulated lighting conditions, was created by Noriega et al. (Wang et al., 2016; Tamir et al., 2017; Mannino et al., 2018; Rojas et al., 2019). Attempts have been made to detect anemia non-invasive, with a focus on the conjunctiva analysis using various methods (Dimauro et al., 2020; Rahman et al., 2020; Suner et al., 2021). However, it is extremely difficult for patients to detect anemia from a conjunctiva image captured with a simple phone camera to be used for communication with the IoT.

The diagnosis of anemia, in non-invasive methods using images of the conjunctiva, palm and nail bed, has its advantages. It also faces some limitations. These limitations include challenges related to accuracy due to variations in skin color, the impact of factors, the presence of medical conditions, and diversity among patients. Additionally, there are concerns regarding data quality, privacy issues costs involved, approval processes, and the need for clinical validation. It is important to remember that any diagnostic approach like this should complement judgment and other tests to ensure reliable results, in real life healthcare settings. To address these limitations effectively requires research, thorough validation procedures, and careful consideration during implementation. Using lip mucosa images for diagnosis offers unique benefits. This method is non-invasive meaning it doesn't cause any pain or inconvenience to patients. It's easily accessible and suitable for affordable screening programs making it patient friendly. By analyzing the lip mucosa, we can avoid the discomfort and possible infection risks associated with blood tests. This approach is particularly appealing to people who have a fear of needles or those who live in healthcare settings, as it promotes acceptance and participation. Further highlighting its potential as a useful and effective diagnostic approach is its adaptability for continuous monitoring and application in certain groups, such as pediatric patients. To guarantee that lip mucosa analysis is helpful in detecting illnesses like anemia, however, it needs be extensively confirmed via scientific research and clinical trials.

In general, machine learning (ML) refers to computer techniques that automatically find approaches and parameters to arrive at the best solution to a problem, as opposed to being pre-programmed by a person to propose a predetermined solution. This learning process is classified as a branch of Artificial Intelligence (AI), which simulates a component of human intellect and can be used for intelligent goals. A crucial component of the ML methodology is the technique used to carry out classification, regression, clustering, or prescriptive modeling. These techniques can be separated into supervised and unsupervised strategies. This research is the first to use lip mucous images for anemia detection in the literature. The aim of this paper is to detect anemia using images of the lip mucous, where the skin tissue is thin, and to confirm the feasibility of non-invasive anemia detection in the home environment. This will be achieved by developing classical ML algorithms trained using the collected patient data and invasive blood values. Six widely used ML algorithms in medical applications, namely artificial neural network (ANN), decision tree (DT), k-nearest neighbors (KNN), logistic regression (LR), naive bayes (NB), and support vector machine (SVM) (Kambeitz et al., 2015; Arbabshirani et al., 2017), were employed to classify the collected data. Subsequently, important statistical metrics were used to evaluate the performance of the algorithms. Participant's data of age between 16 and 76 was used and just confirmed anaemia cases as well were chosen. Using the lips moucas images, for diagnosis. Two extracted features from lip moucas images (RGB and HSV), age, sex and haemoglobin level, the experiment was done to predict anaemia using machine learning algorithms.

The accuracy of anemia diagnosis based on lip mucosa analysis can be significantly impacted by a variety of factors related to lip appearance and conditions, including lip pallor caused by vitamin B12 deficiency, dark spots, uneven skin tone, and abnormalities like scaly or thick lips, lip sores, or leukoderma. These elements may alter the hue or create distortions in the pictures of the lips, which may cause misunderstandings. The development of reliable algorithms that take into consideration these lip-related alterations and distinguish between true anemia-related color changes and those resulting from other lip disorders is essential for effective anemia identification. Furthermore, to reduce the possibility of misdiagnosis and guarantee accurate findings, clinical judgment and a comprehensive patient evaluation should be used in conjunction with computerized diagnosis. The examination of the lip mucosa for the diagnosis of anemia has obstacles from smoking and the use of lipstick, which requires identifying possible problems with changes in lip color, changes in texture and the variability of data due to these variables. The data set must be supplemented with photos of smokers and lipstick users, and machine learning algorithms must be modified to account for these particular traits. These steps are necessary to improve accuracy and sensitivity. Even in populations with different lip diseases caused by smoking or lipstick usage, the reliability and usefulness of the diagnostic procedure can be guaranteed by carefully weighing these difficulties and implementing mitigation measures.

The study is important due to its utilization of AI through ML, which is widely used for medical prediction and diagnosis (Kononenko, 2001; Jiang et al., 2017; Christodoulou et al., 2019; Alballa and Al-Turaiki, 2021). Furthermore, this study offers a non-invasive method that is more convenient and accessible to patients than blood tests, especially in areas where these tests are not readily available. Early detection of anemia is crucial as it can prevent serious health consequences such as fatigue, weakness, and decreased immune function. Moreover, developing and training ML algorithms to analyze lip mucous images is a cost-effective screening method in areas where healthcare resources are limited. Furthermore, the objective analysis of lip mucous images by ML algorithms reduces the risk of human error or bias in the diagnosis of anemia. Lastly, the ML algorithm could be used to quickly and accurately analyze large volumes of lip mucous images, which can potentially increase the efficiency of anemia screening programs once validated. In comparison to previous research and conventional diagnostic techniques, the approach outlined in our work shows potential for greatly improving the accuracy and sensitivity of anemia identification. The use of machine learning algorithms with features selection, the inclusion of different data kinds and demographic data, a sizable and diverse dataset, and thorough evaluation utilizing performance measures are all credited with this increase. Furthermore, lip mucosa analysis's non-invasiveness, affordability, accessibility, and capability for continuous monitoring should result in improved patient compliance and faster anemia identification. Though rigorous validation, comparisons with other diagnostic techniques, and clinical trials are essential stages in proving its superiority in actual clinical practice, they are not sufficient by themselves to support these assertions.

The remaining parts of the article are planned as follows: Section 2 provides a review of anemia detection using non-invasive methods. Section 3 explains the methodology. Section 4 outlines and presents the results. Section 5 presents the discussion. The last section concludes the article and suggests directions for future research.

## Related work

In the field of biomedical data classification, numerous studies have been published in the last decade, providing a foundation for the current research. This section will thus focus on the details of previously applied non-invasive methods for anemia detection, including conjunctiva and fingertip analysis, as well as the ML models employed in these studies.

Suner et al. aimed to detect anemia in patients in the emergency room at a training and research hospital (Suner et al., 2021). In the first stage, images of both conjunctiva of 142 patients were taken with a smartphone. From each image, a region of interest was selected that targeted the palpebral conjunctiva. Image-based parameters were extracted and used in step-by-step regression analyses to develop a predictive model of predicted hemoglobin (HBc). In phase 2, a validation model was created with data from 202 new emergency room patients. The final model, based on all 344 patients, was tested for accuracy of anemia and transfusion thresholds.

Rojas et al. designed an application called selfienemia to estimate hemoglobin levels under controlled lighting conditions (Rojas et al., 2019). After taking a photo and processing it in the app, a colorimetric analysis is performed using a mathematical model from the cloud service. A special camera was used outside the application for better control of external conditions in this prototype. Sixty-four tongue images and 64 conjunctival images were taken, and the results of the application were compared with traditional large blood count (CBC), which is considered the gold standard test for diagnosing anemia. In the analysis of tongue images, the results were 91.89% sensitive and 85.18% specific, and in the analysis of palpebral conjunctiva, the results were 91.89% sensitive and 70.34% specific.

Sevani et al. aimed to support the process of detecting anemia using conjunctival pallor with a smartphone camera (Sevani et al., 2018). They applied the K-Means clustering method to analyze the pixels of conjunctival images represented by digital characters in RGB formats. They compared the test results obtained from this application with laboratory results, demonstrating that the method provided an accuracy of 90%. Mannino et al. estimated hemoglobin levels by analyzing the color and metadata of nail bed photos taken with a smartphone (Mannino et al., 2018). In their study of 100 people, they were able to test anemia with a sensitivity of 97%.

Hasan et al. utilized video images of fingertip captured with a smartphone camera under a flashlight and trained ANN to predict HGB levels non-invasively (Hasan et al., 2018). Red, green, and blue pixel densities were calculated in 100 blocks of fields in each frame in 10 seconds (300 frames) of recorded video by 75 adults, and this method was applied to all 300 frames. ANN was then used to develop a derived model for predicting hemoglobin levels. They found that there was a correlation of 0.93 between the model and gold standard hemoglobin levels in their sample of patients aged 20 to 56 years.

Tamir et al. developed an android application to capture a photo of the anterior eye conjunctiva with a smartphone camera in suitable lighting conditions with appropriate resolution (Tamir et al., 2017). These images were then processed to obtain spectra of the conjunctival color and RGB components, which were compared to a threshold to determine anemia. In their study of 19 subjects whose hemoglobin levels were known, they compared the values of 15 people with the laboratory results and correctly identified them at a rate of 78.9%.

Li et al. proposed a novel method with dynamic spectrum, using a spectrograph with a computer to scan the transmission spectrum of fingertip (Li et al., 2017). An average prediction correlation coefficient (R) of 0.8399 is achieved in their experiment. Using a light source, Wang et al. performed chromatic analysis of images taken of the patient's finger, using an application called HemaApp (Wang et al., 2016). When they analytically evaluated 31 patients between the ages of 6 and 77, they obtained a correlation of 0.82 with the blood test. HemaApp showed a sensitivity of 85.7% and a specificity of 76.5% in anemia screening.

Most previous methods for detecting anemia have utilized data such as conjunctival images, human nails, and fingertip. ML has been used in some of these methods to develop both invasive and noninvasive approaches for detecting anemia. However, there is still a need to improve the performance of these methods by incorporating new data and employing more straightforward approaches. This study is particularly important as it is the first to utilize lip mucous images for predicting anemia using ML. The study's use of ML techniques, which has been widely employed in the medical field for prediction and diagnosis (Kononenko, 2001; Jiang et al., 2017; Christodoulou et al., 2019; Alballa and Al-Turaiki, 2021), is what provides it with its significance.

## Methodology

The classification problem aims to detect anemia using a dataset of collected lip images. The study begined by building a dataset that includes two types of lip images: healthy and anemic. Data preparation was then performed, followed by the using of ML models for classification, which were evaluated to determine the best model.

###  Participants

Following the Sakarya University Ethical Approval (E-71522473-05.01.04-74571-458), participants were recruited between November and December 2021. The study group consisted of participants residing in the Pamukova District of Sakarya Province. Data were collected from 138 participants, including 100 women and 38 men by a team of experienced medical doctors. Smokers images have not been included in this study. According to the WHO criteria, 23 women and 6 men were diagnosed with anemia (Organization, 2011). The range of hemoglobin level 129 level of healthy individuals was 121 to 167 grams per liter (g/L). The study group was selected using the convenience sampling method (Patton, 2005). The range of haemoglobin level for male was 133–167 g/L with ages 16–68 years old between and the range of haemoglobin level for female was 121–158 g/L with ages between 11 and 76 years old. The range of anemia patient was 80–130 grams per liter (g/L). Demographic variables such as gender and age ranges are presented in Table 1.

**Table 1:** Participants demographic values.

**Variable**		**Frequency**		**Percentage of anemia patients**
		**Healthy**	**Anemia**	
Gender	Female	77	23	72.46
	Male	32	6	27.54
	18–30	53		38.4
Age	30–50	36		26
	50	49		35.6

### Experimental Setup

**Figure 1:**
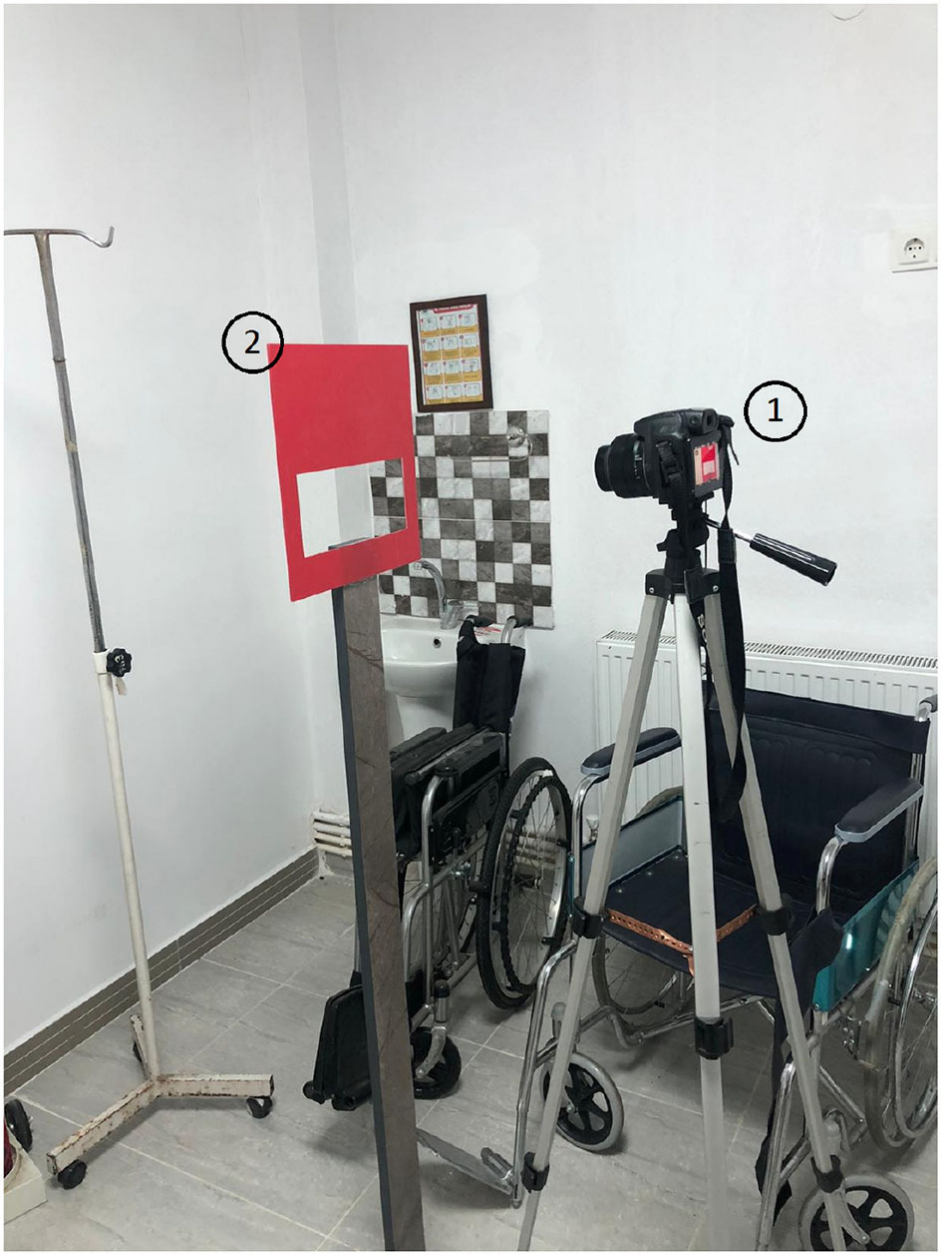
System setup: (1) camera, (2) frame.

**Figure 2:**
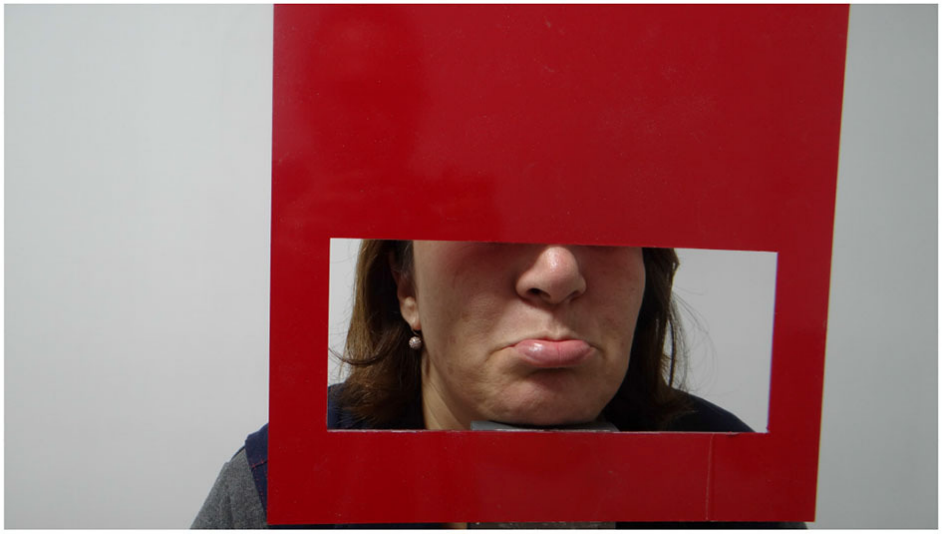
Healthy person representation.

**Figure 3:**
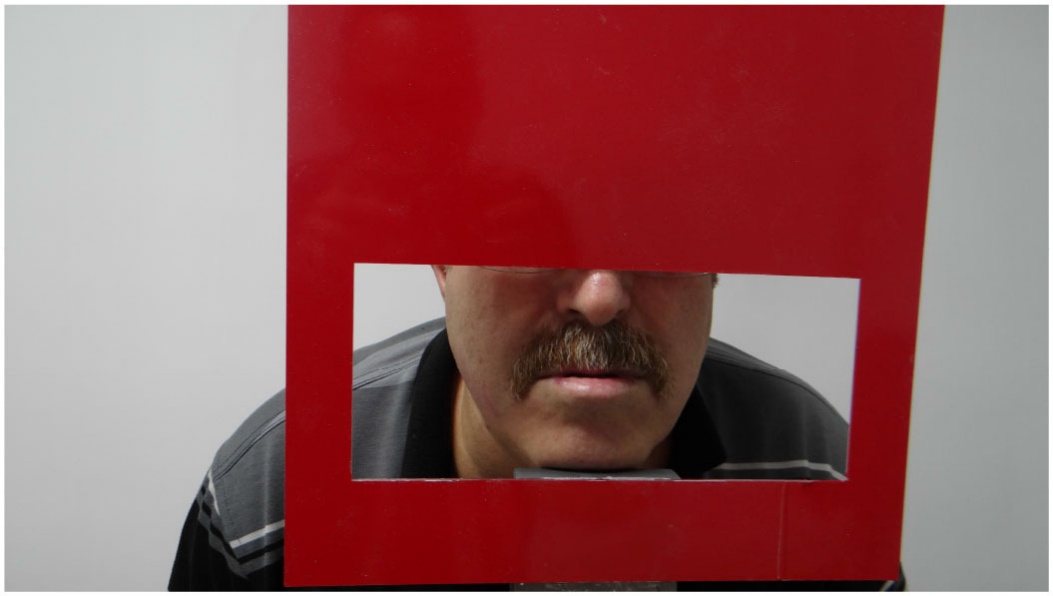
Anemia patient.

The experimental setup shown in Figure 1 was designed to measure the facial features of the participants, it is designed to display only the lip area with the help of an adjustable frame. A Canon camera EOS 2000D was used for collecting the lip moucas images. The camera's flash was used for lighting. Examples of collected lip mucosa images for healthy and non healthy one are shown in Figures 2, 3 respectively.

### Software Setup

**Figure 4:**
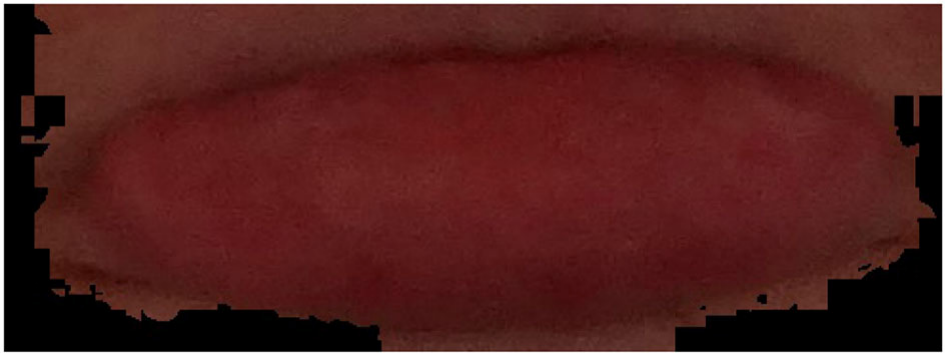
Healthy lips pallor with Hb of 137 grams per liter (g/L).

**Figure 5:**
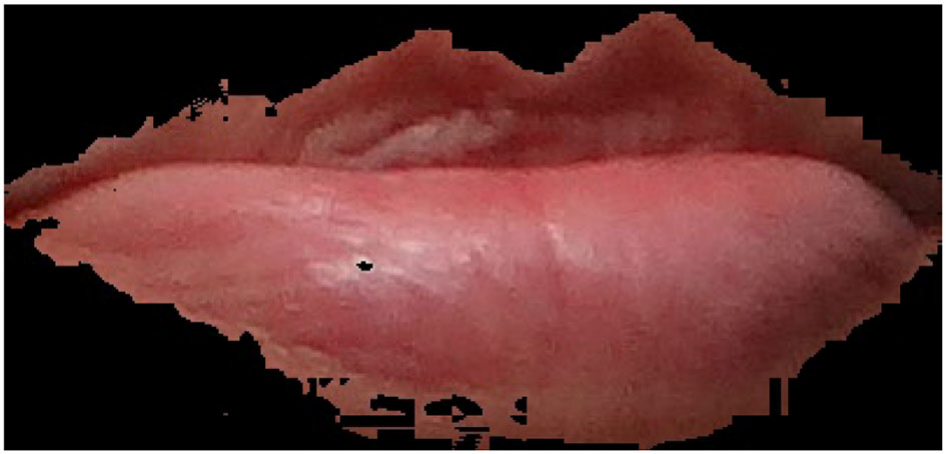
Anemia patient lips pallor with Hb of 116 grams per liter (g/L).

A custom Python application was developed to process and analyze the data collected from the participants. Firstly, the lip contour of each participant was obtained using corner detection, thresholding, and framing (see Figures 4, 5 for lips pallor examples). Next, the digital image within the frame was converted to RGB and HSV formats. Finally, classical ML algorithms were employed to perform the classification task. These steps will be discussed in detail in this section.

### Data processing and preparing

**Figure 6:**
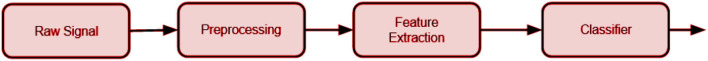
Schematic of data processing and ML application.

Feature extraction and classification of the images and hemoglobin data obtained using the setup are explained in Figure 6. A high-resolution image (4896x2752) of the participants was captured using a camera. The images were collected on the same day as the blood sampling. Thus, the data duration and the number of samples taken from each subject are close but not the same. Two different data types were obtained from participants' images as features: RGB (Red, Green, Blue) red color values and HSV saturation values, along with age, sex, and hemoglobin levels, which were used for classification. Feature extraction identified different features of the image that were sent to the classification algorithm, thereby increasing the classification success.

### Data Normalization

In the absence of normalization adjustments, the larger scale variable will totally dominate ML algorithms' attempts to predict trends. Numerous ML methods demand statistical rescaling of their input variables to prevent sacrificing numerical stability (Trentin, 2015). In order to improve the model's fit to the supplied data, minimum maximum normalization techniques are used (Senvar and Sennaroglu, 2016). According to it, all attributes are equally important in terms of size (Singh and Singh, 2020). The unnormalized 145 data are linearly adjusted by the normalization technique to a defined lower and upper bound (Han et al., 2011). Typically, the dataset is rescaled to lie between 0 and 1 or -1 and 1. In this study, the minimum maximum normalization technique with a [0,1] scale was examined. The five inputs were normalized to the value between [0 and 1]. Equation 1 demonstrates the process used to transform raw data into normalized data, where X stands for the real data, *X*_*min*_ represents the lowest value found in the dataset of all X values, and *X*_*max*_ reflects the highest X-value found in the dataset. Thus, *X*_*normalized*_ displays the normalized X value and ranges from 0 to 1.


(1) $$\begin{array}{l} X_{normalized}=X-X_{min}/X_{max}-X_{min} \end{array}$$

### Machine Learning Algorithms

DT is a decision-making method that has a tree structure and Random Forest (RF) is an ensemble classifier that consists of many decision trees and outputs the class that is the mode of the class's output by individual trees. It improves predictive accuracy with average and reduces over fitting (Gnanapriya et al., 2010; Schonlau and Zou, 2020; Mansour et al., 2023). In this study, the DT were constructed with a maximum depth of 1 for estimating the three joints moment. SVM is a popular supervised ML algorithm used for both classification and regression tasks. It is particularly effective in solving complex problems with high-dimensional data. SVM aims to find an optimal hyperplane that separates the data points of different classes, maximizing the margin between the classes.

For supervised classification and regression applications, the KNN algorithm is a non-parametric, instance-based learning technique. KNN is adaptable and useful for a variety of tasks because, unlike many other machine learning algorithms, it does not make firm assumptions about the distribution of the underlying data. The foundation of KNN is the idea that data points with comparable properties are more likely to fall into one class or display similar goal values. For supervised classification problems, the NB algorithm is a probabilistic ML technique. Although NB is straightforward and makes the “naive” premise of feature independence, it has been successful in a number of fields. In order to shed light on the NB algorithm's inner workings and demonstrate its adaptability for practical applications. A popular linear model in ML for binary and multi-class classification applications is LR. Despite being straightforward, it acts as a crucial building element for a number of sophisticated procedures.

**Figure 7:**
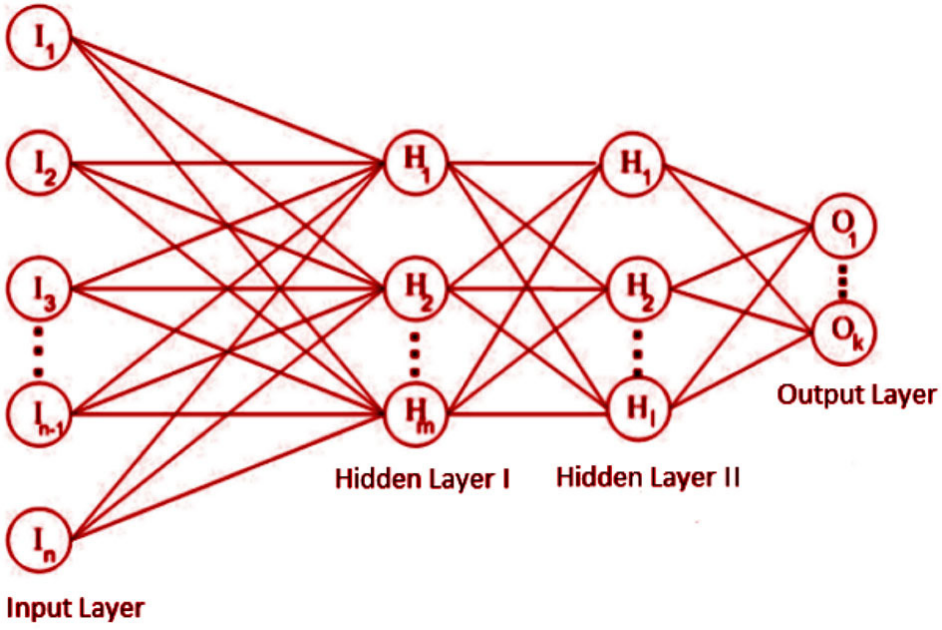
Feed-forward ANN.

Compared with previous ML techniques, ANN algorithm is widely applied for biomedical data classification. As lack of large data sets in the healthcare and interconnected complex relationships between the individual biological components have encouraged the scientific research community to integrate ANN models. It adapt within nonlinear boundaries and is efficient to provide better classification. ANN has the ability to learn from continuous data and update the model, which is not present in other ML algorithms such as the decision tree. An example of a back feed-forward neural network is shown in Figure 7. It is a simple classification algorithm where the information is routed from input to output. The backpropagation algorithm was a widely utilized technique for training multiple-layer perceptrons. The multilayered layer perceptron's weight values are adjusted by approaching the intricate intersection of input and output data. The structure of the model in this study was obtained when the input, output, and one hidden layer are 10-5-1 (number of neurons) Input, hidden, and output layers, respectively, used Relu, Relu, and sigmoid activation functions. The ANN model were applied with various hidden layer neurons utill it reaches its best results, it was trained from 5 to 10 neurons. The training cycle was 100 and Adam optimizer was used.

The performance of ML algorithms is critical to their usefulness and effectiveness in solving real-world problems (Hossin and Sulaiman, 2015; Santafe et al., 2015; Mansour et al., 2023). High-performing algorithms can make more accurate predictions, process data more efficiently, scale to handle large datasets, and be more interpretable, leading to better decision-making and improved outcomes. Classification models are widely used in ML to predict outcomes based on a set of characteristics. To evaluate the performance of such models, several commonly used metrics are available. The choice of metric depends on the nature of the problem, class balance, and desired outcome of the model (Prati et al., 2011; Santafe et al., 2015; Tharwat, 2021). Accuracy is a simple metric that measures the proportion of correct predictions out of all predictions made by the model (Folorunso et al., 2022). However, it may not be the best choice when there is class imbalance in the data. Precision measures the proportion of true positives out of all positive predictions made by the model and is useful when the goal is to minimize false positives (Juba and Le, 2019; Miao and Zhu, 2022). Recall, on the other hand, measures the proportion of true positives out of all the actual positive examples in the data, and is useful when the goal is to minimize false negatives (Ali et al., 2022; Miao and Zhu, 2022). F1 score is the harmonic mean of precision and recall and is useful to balance the importance of both (Hossin and Sulaiman, 2015). AUC measures the ability of the model to distinguish between positive and negative examples and is useful when identifying the best threshold to separate positive and negative examples (Tharwat, 2021). Finally, the confusion matrix provides a more detailed view of the performance of the model than any single metric by displaying the number of true positives, true negatives, false positives, and false negatives for the given model (Haghighi et al., 2018; Liang, 2022). Overall, the performance evaluation of a ML model is essential to determine its effectiveness and identify areas for improvement. A combination of metrics should be used to evaluate the model, and the context of the problem should be considered when choosing a metric.

## Results and discussion

**Figure 8:**
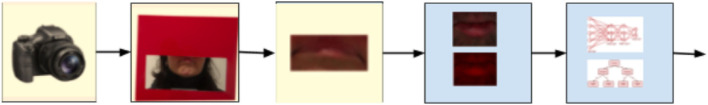
Flowchart of data classification.

The experiments were performed using the Python programming language and its Keras and Tensorflow libraries. This research classifies anemia by applying the input characteristics produced with the extraction of RGB (Red value), HSV (Saturation), age, gender, hemoglobin levels. In the classification process, the spilt data function was applied to separate the data into train, val and test data where 60% (82) of the dataset was randomly assigned as train data, 20% (28) were randomly assigned as validate and 20% (28) as test data. The flow diagram describing the processing and classification is shown in Figure 8, where the process starts with the collection of patient data and images, the cropping of the image, feature extraction and the preparation of the data, and finally classification and prediction of anemia cases and evaluation of ML models. In the classification process, six algorithms were applied; KNN, NB, LR, SVM, DT, and ANN algorithms, which are frequently applied in the anemia detection literature, were used. To evaluate the classification algorithms used in this study; different methods were used. These methods are accuracy, precision, recall, and F1 score.

**Figure 9:**
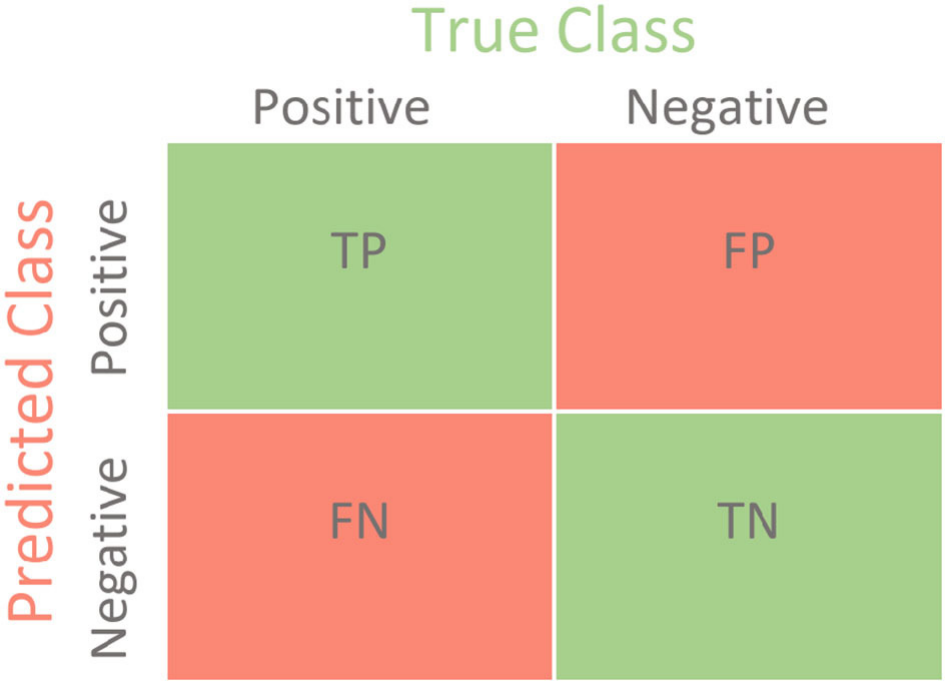
Confusion matrix.

**Figure 10:**
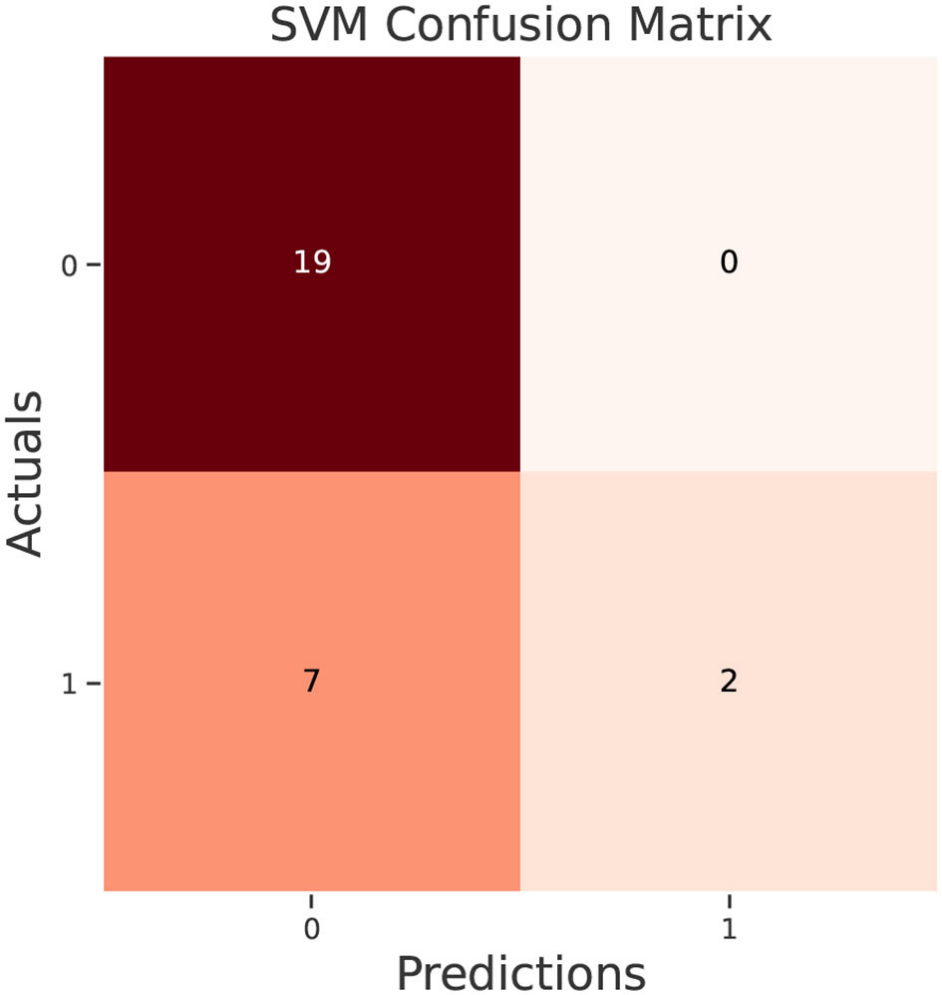
SVM confusion matrix.

**Figure 11:**
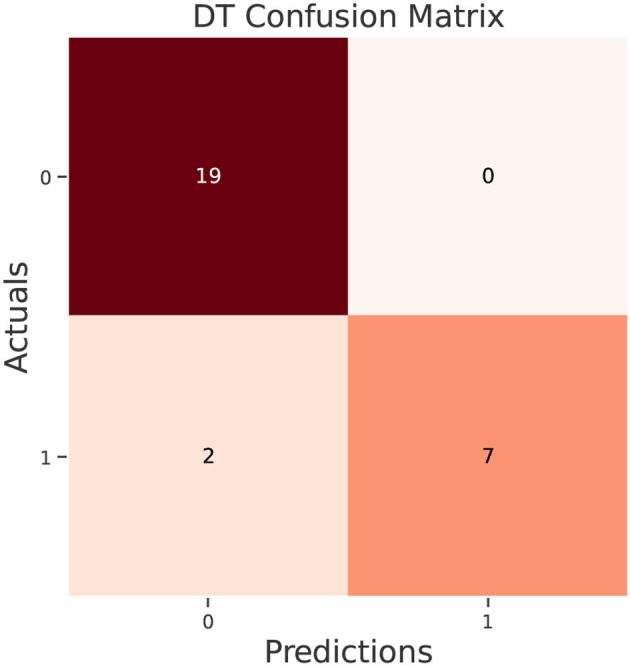
DT confusion matrix.

**Figure 12:**
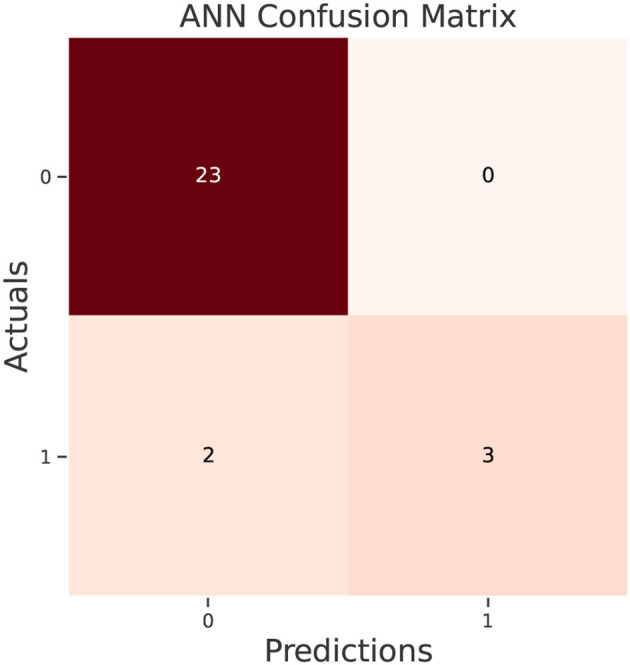
ANN confusion matrix.

**Figure 13:**
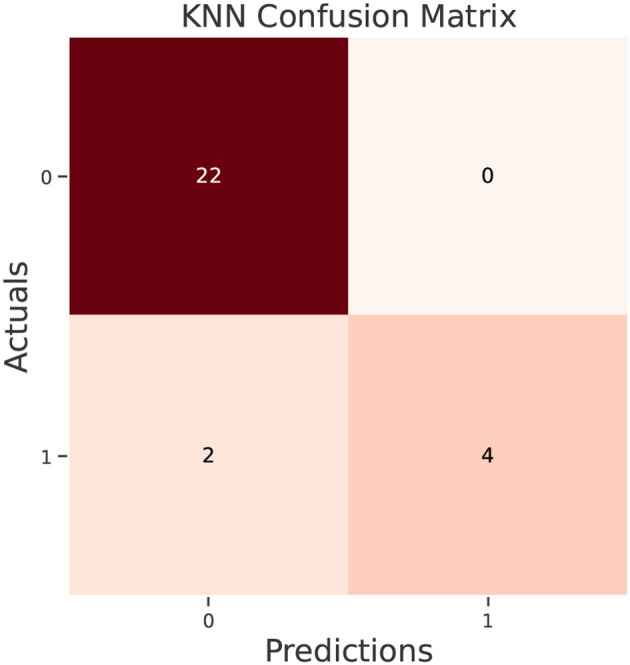
KNN confusion matrix.

**Figure 14:**
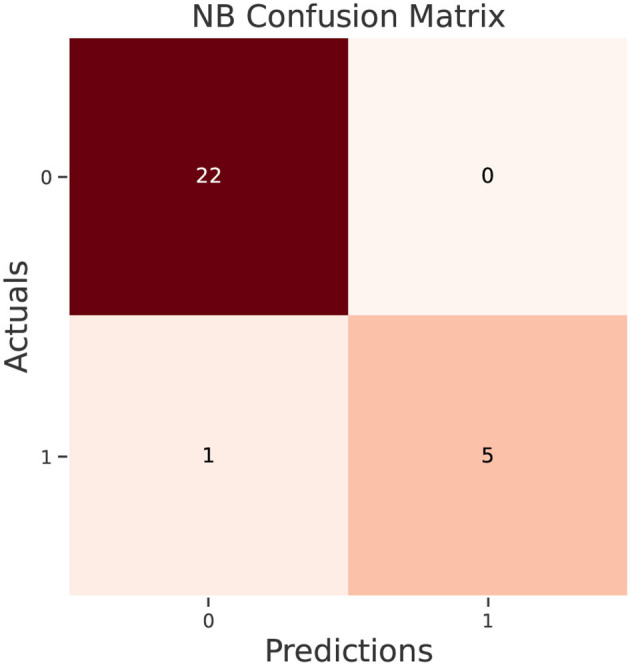
NB confusion matrix.

**Figure 15:**
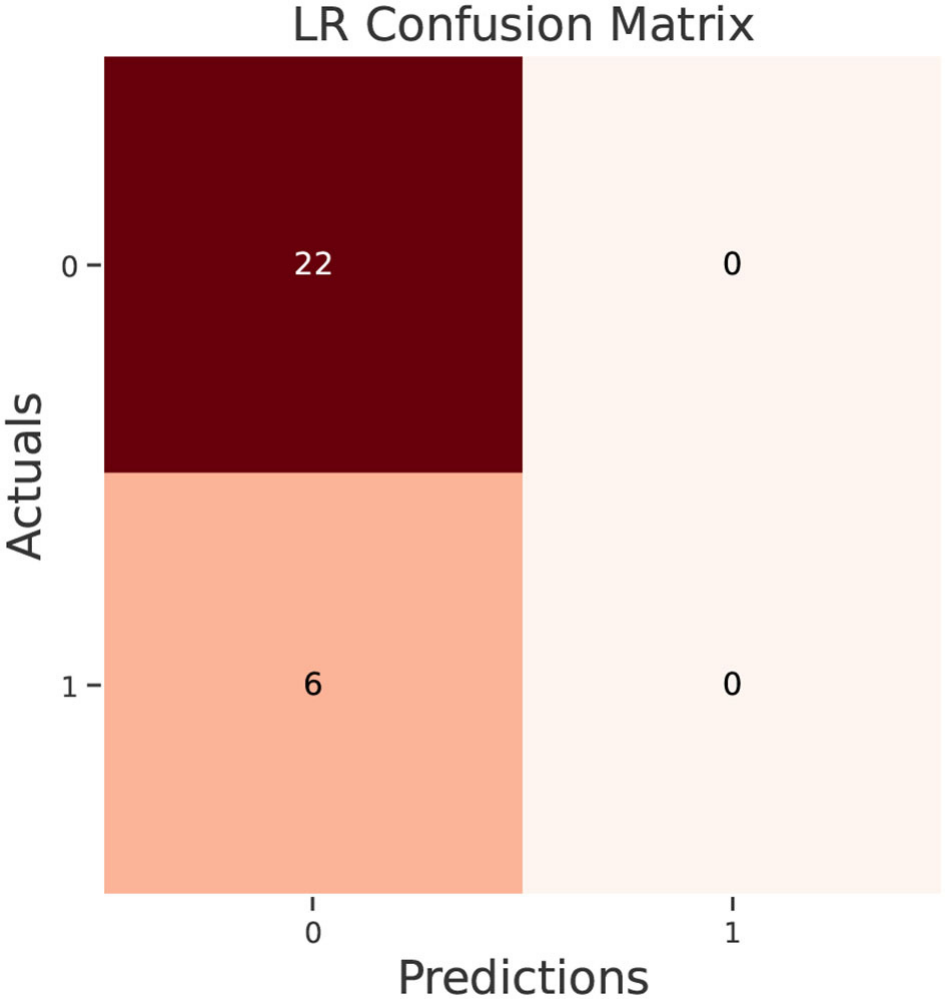
LR confusion matrix.

The test data confusion matrix prediction evaluation for SVM, DT, ANN, KNN, NB and LR are shown in Figures 10–15. Four main points to consider here, True positive (TP), False Positive (FP), True Negative (TN) and False Negative (FN). These points clarify the success of the algorithm where TP and TN are the correct predictions in the both positive and negative classes, and FP and FN are the false predictions respectively (Figure 9). For SVM algorithm (Figure 10), the TP was 19 and the TN was 2 from a total of 19 and 9 respectively. Figure 11 presents the confusion matrix for DT algorithm. For DT, the TP was 19 and the TN was 7 from a total of 19 and 9 respectively. Figure 12 presents the confusion matrix for ANN algorithm. For ANN, the TP was 23 and the TN was 3 from a total of 23 and 5 respectively. Figure 13 presents the confusion matrix for KNN, the TP was 22 and the TN was 4 from a total of 22 and 6 respectively. Figure 14 presents the confusion matrix for NB, the TP was 22 and the TN was 5 from a total of 22 and 6 respectively. Finally, Figure 15 presents the confusion matrix for LR, the TP was 22 and the TN was 0 from a total of 22 and 6 respectively.

The success rates of all algorithms are shown in Tables 2–5 where Tables 2, 3 are the evaluation of both train and validation data. Tables 4, 5 present the macro and weighted average of the test data for both the positive and negative classes. In these tables and based on confusion matrices, four main parameters; accuracy (Equation 2), AUC (area under the ROC curve), precision (Equation 3), recall (Equation 4) and F1 score (Equation 5) which evaluate the six algorithms applied to classify anemia applying the input characteristic matrix of RGB (red value), HSV (saturation), age, sex, hemoglobin levels.


(2) $$\begin{array}{l} Accuracy=\frac{TP+TN}{TP+TN+FP+TN} \end{array}$$

(3) $$\begin{array}{l} Precision=\frac{TP}{TP+FP} \end{array}$$

(4) $$\begin{array}{l} Recall=\frac{TP}{TN+FN} \end{array}$$

(5) $$\begin{array}{l} F1=\frac{2*Precision*Recall}{Precision+Recall} \end{array}$$

**Table 2:** Train data; accuracy, precision, recall and F1 score.

**Algorithm**	**Accuracy (%)**	**Precision**	**Recall**	**F1 score**
SVM	84	42	50	46
LR	93	96	82	87
DT	100	100	100	100
ANN	79	40	50	44
KNN	90	91	79	83
NB	93	96	82	87

**Table 3:** Val data; accuracy, precision, recall and F1 score.

**Algorithm**	**Accuracy (%)**	**Precision**	**Recall**	**F1 score**
SVM	75	38	50	43
LR	96	98	92	94
DT	93	86	96	89
ANN	75	38	50	43
KNN	89	86	81	83
NB	96	98	92	94

**Table 4:** Test data macro averages; accuracy, precision, recall and F1 score.

**Algorithm**	**Accuracy (%)**	**AUC**	**Precision**	**Recall**	**F1 score**
SVM	75	86	87	61	60
LR	79	50	93	50	44
DT	93	95	89	95	91
ANN	93	96	96	80	85
KNN	93	83	96	83	88
NB	96	91	98	92	94

**Table 5:** Test data weighted averages; accuracy, precision, recall and F1 score.

**Algorithm**	**Accuracy (%)**	**AUC**	**Precision**	**Recall**	**F1 score**
SVM	75	86	87	61	60
LR	79	50	62	79	69
DT	93	95	94	93	93
ANN	93	96	93	93	92
KNN	93	83	93	93	92
NB	96	91	97	96	96

When comparing the results obtained from the anemia classification performed using ML techniques, it was found that the disease was diagnosed with an accuracy success rate of 96% using NB, 93% using DT KNN and ANN, LM classifies the data with an accuracy success rate of 79% and SVM classifies the data with an accuracy success rate of 75%. The AUC were 50, 83, 86, 91, 95, and 96 for LR, KNN, SVM, NB, DT, and ANN respectively. Although this value seems high, higher percentages and more data-trained models are required to use it as a medical diagnosis.

## Discussion

In this study, extracted data from images of lip mucous were used to train a ML models to identify anemia. The results of this study are in agreement with those of previous investigations that used ML models to predict anemia using both invasive and non-invasive techniques. Accuracy, precision, recall, and F-score were used to assess how well ML models performed in predicting anemia. The models examined demonstrated high and considerable accuracy. For predicting anemia, NB reported the highest accuracy, the highest precision and F1 score and SVM reported the lowest scores.

Limited methods have been found in the literature to detect anemia using ML. For example, the K-means clustering technique was used to carry out conjunctival pallor image-based anemia detection, which demonstrated an accuracy of 90% when comparing the test results acquired from this application with laboratory results (Sevani et al., 2018). ANN were used to assess images of fingertip captured with a smartphone camera to predict HGB levels non-invasively. As a result, a correlation of 0.93 was observed between the model and gold standard hemoglobin levels (Hasan et al., 2018). Convolutional neural networks was techniques was used to carry out conjunctival image-based anemia detection with accuracy of 94% (Magdalena et al., 2022). YOLO v5 was used to detect anemia using conjunctiva image collection with sensitivity of 71% and a specificity of 89% (Rivero-Palacio et al., 2022). AlexNet was used to calculate total hemoglobin concentration by developing frequency-domain multidistance approach, based on a non-contact oximeter, provided data on total hemoglobin with accuracy of 87.50% (Moral and Bal, 2020). Compared to these methods that used ML in general for anemia detection, the current study presents a non-invasive method that uses ML models to detect anemia with higher accuracy, reaching 99% using DT. This new method is simple and can be developed for the detection of real-time anemia. Several methods were found in the literature to detect anemia.

Anemia is a prevalent condition that affects millions of people worldwide, but it often goes undiagnosed until it becomes severe. Early detection of anemia is crucial for effective treatment, which is why the use of ML to detect anemia through lip mucosa image classification could be significant. This method provides a non-invasive and cost-effective alternative to traditional anemia screening methods such as blood tests, which can be invasive and costly, particularly in resource-limited settings. The automation of diagnosis through ML algorithms can reduce the need for expert human intervention and speed up the process of diagnosis and treatment. The potential for automation also makes this method scalable and easily accessible, allowing for widespread implementation of anemia detection tools. Our research suggests that employing ML approaches to detect anemia will aid in classifying the diagnosis, which will then help in the creation of efficient preventive measures. As a result, this research evaluates the predictive capability of several ML algorithms in addition to addressing the integration of cutting-edge technology for the prediction and diagnosis of low hemoglobin levels.

When compared to other human body parts, lip pallor, which is defined by the paleness or loss of natural color in the lips, provides significant advantages as a non-invasive diagnostic site. First of all, there is no need for specialist equipment or invasive procedures to examine the lips because they are quite obvious and accessible. Due to its accessibility, lip pallor is a sensible option for diagnostic techniques, enabling quick and little disruptive patient examinations. A rich vascular network with countless small blood vessels close to the surface is also present on the lips. Due to the vascular richness, variations in blood flow and oxygenation, which frequently show up as changes in lip color, may be quickly identified. The lips are perfect for identifying blood-related diseases like anemia because of the strong relationship between lip color and the circulatory system. This provides real-time information about a patient's health.

Lip pallor analysis is non-invasive, which increases patient comfort and compliance. The procedure is harmless and acceptable for people of all ages, whether it involves straightforward eye exams or more sophisticated imaging techniques. This technique also fits well with ethical standards and cultural norms because it is typically socially acceptable in all cultures to examine one's lips. Such acceptance may increase a patient's willingness to participate in lip-based diagnostic tests. The ability to capture high-resolution photographs of the lips thanks to advancements in imaging technology also makes it possible to analyze color variations and texture changes precisely. This degree of specificity is essential for identifying minute symptoms of diseases like anemia and guarantees that lip pallor analysis will always be a practical and affordable screening technique in medical settings, making it a crucial tool in the field of non-invasive diagnostics.

The contribution of this study to medical research is also significant, as the use of ML in medical research is still in its early stages. This study could provide a foundation for further research and development of ML based tools for anemia detection and diagnosis, potentially leading to even more accurate and effective diagnostic tools. The use of ML for anemia detection using lip mucosa image classification could have significant implications for healthcare, particularly in resource-limited settings where traditional screening methods may not be readily available. The potential for early detection, non-invasive and cost-effective screening, automation of diagnosis, contribution to medical research, and further development of diagnostic tools make this study a promising avenue for improving healthcare outcomes.

## Conclusions

Images of the lip mucosa, which have thin skin tissue, were used in this study to identify anemia. Data from 138 patients, including 100 women and 38 men, were collected. Rgb red color values and hsv saturation values were obtained from participant images and used as features, along with age, sex, and hemoglobin levels, to perform classification. The efficacy of ML models in predicting anemia was tested using accuracy, precision, recall, and F score. The findings indicated that among the ML algorithms utilized, NB achieved the highest accuracy at 96%, while SVM received accuracy ratings of 75%. This research suggests that using ML to recognize anemia will aid in classifying the diagnosis, which would subsequently facilitate the development of effective preventive measures.

This work has established a link between anemia detection and lip mucous images, where the conjunctiva is used in general practices. This newly found method is easy to use, and it was demonstrated for the first time that it can be classified on the lip mucous. Although the accuracy of 93% appears high, more data-trained models and larger percentages are needed to use it as a diagnostic in medicine. In the future, augmented data will be used for an online classification along with deep neural networks and a mobile application.

## Data availability statement

The raw data supporting the conclusions of this article will be made available by the authors, without undue reservation.

## Ethics statement

The studies involving humans were approved by Sakarya University Ethical Approval (E-71522473-05.01.04-74571-458). The studies were conducted in accordance with the local legislation and institutional requirements. The participants provided their written informed consent to participate in this study.

## Author contributions

TD colleting the lip mucosa images and literature review. MM preparing the software and manuscript writing. MK preparing the images and feature extraction. CF writing the manuscript. SM writing the manuscript. All authors contributed to the article and approved the submitted version.
