# Corrigendum: Anemia detection through non-invasive analysis of lip mucosa images

**DOI:** 10.3389/fdata.2023.1335213

**Published:** 2023-12-11

**Authors:** Shekhar Mahmud, Turker Berk Donmez, Mohammed Mansour, Mustafa Kutlu, Chris Freeman

**Affiliations:** ^1^Department of Systems Engineering, Military Technological College, Muscat, Oman; ^2^Department of Biomedical Engineering, Sakarya University of Applied Sciences, Serdivan, Sakarya, Türkiye; ^3^Department of Mechatronics Engineering, Sakarya University of Applied Sciences, Serdivan, Sakarya, Türkiye; ^4^Electronics and Computer Sciences, University of Southampton, Southampton, United Kingdom

**Keywords:** anemia, machine learning, classification, support vector machine (SVM), decision tree

In the published article, there was an error in the author list.

The new requested author order is: Shekhar Mahmud^1^, Turker Berk Donmez^2^, Mohammed Mansour^3*^, Mustafa Kutlu^3^ and Chris Freeman^4^

^1^ Department of Systems Engineering, Military Technological College, Muscat, Oman

^2^ Department of Biomedical Engineering, Sakarya University of Applied Sciences, Serdivan, Sakarya, Türkiye

^3^ Department of Mechatronics Engineering, Sakarya University of Applied Sciences, Serdivan, Sakarya, Türkiye

^4^ Electronics and Computer Sciences, University of Southampton, Southampton, United Kingdom

The authors apologize for this error and state that this does not change the scientific conclusions of the article in any way. The original article has been updated.

